# Lumbar Puncture, a Direct Method for the Treatment of Tetanus with the Report of Nine Successful Cases so Treated

**Published:** 1904-12

**Authors:** W. Troy Bivings

**Affiliations:** Atlanta, Ga.


ATLANTA

### Journal-Record of Medicine.

Successor to Atlanta Medical and Surgical Journal, Established 1855, and Southern Medical Record, Established 1870.

OWNED BY THE ATLANTA MEDICAL JOURNAL CO.

Published Monthly.

VOL. VI. DECEMBER, 1904. No. 9.

BERNARD WOLFF, M.D., EDITOR, Nos. 319-20 Prudential.

M. B. HUTCHINS, M.D., BUSINESS MANAGER, 1007-1008 Century Bldg.

J. N. LECONTE, M.D., ASSOCIATE EDITOR.

### ORIGINAL COMMUNICATIONS.

### LUMBAR PUNCTURE, A DIRECT METHOD FOR THE TREATMENT OF TETANUS WITH THE REPORT OF NINE SUCCESSFUL CASES SO TREATED.*

BY W. TROY BIVINGS, A.B., PH.B., M.D.,

ATLANTA, GA.

Tetanus, or lockjaw, as it is more commonly known, is an infectious disease, caused by the lodgment in a wound or some mucous surface of a specific micro-organism known as the bacillus Nicolaier. It is one of the smallest bacilli yet discovered, and is a spore-producing organism, the spore occurring only in one end of the rod, thus giving it the appearance of a small pin or drumstick. It will not grow in the presence of oxygen or carbon dioxide gas, but grows very nicely in the atmosphere of pure hydro-

*Read before the Hospital Graduates Medical Club of Atlanta, September 13, 1904.

gen. Is found in nature as an inhabitant of the soil even to the depth of several feet, in manure, old masonry, garden earth, etc., and it is said certain savages of the New Hebrides poisoned their arrow-heads with tetanus by smearing them with mud from crab-holes in the marshes. The spores of the tetanus bacillus are very hard to kill. They retain their vitality for years and years in a dry state, and will stand an exposure of one hour to eighty degrees C, but only last five minutes when exposed to one hundred degrees C, in a steam sterilizer. Some animals are more susceptible to the toxines of tetanus than others. Park says "that an amount of toxine sufficient to kill a hen would suffice to kill five hundred horses."

A fact of the greatest importance in the treatment of traumatic tetanus is that a mixed infection is necessary to the development of the disease, when the infection is produced by spores, which is almost invariably the case. Given a wound of the hand, parts healthy, little or no bruising, the saprophytic germs are rapidly destroyed by proper antiseptics, no mixed infection occurs and tetanus fails to develop, while with a bruised and lacerated wound where a mixed infection has occurred tetanus is very liable to make its appearance.

To Nicolaier, Kitasato, Catani, Tizzoni and Behring we are indebted for the antitetanic serum, which theoretically should have been a specific, but practically lowered the death-rate but little at all when used subcutaneously. The failure of this method of procedure induced Roux and Borrell to attempt the intracerebral injections of the antitetanic serum, first having removed a button of bone by means of the trephine. The results, however, obtained were far from satisfactory, and after repeated trials by some of our best surgeons the injection of antitetanic serum into the lateral ventricles was discarded; even Hartley, of New York, who was one of the most ardent supporters of this method for the treatment of tetanus, has long since abandoned the same. Frequent attempts have been made by German, French and American investigators to treat tetanus by means of spinal injections of antitetantic serum, but the results obtained have been disappointing, and the mortality but little reduced.

In regard to the action of the tetanus toxines in the body, there seems to be great differences of opinion among the writers on this subject. According to Gumprecht, the action of tetanus depends on an increased reflex of excitability, as in strychnine poisoning; but it is different from strychnine in its mode of distribution, and probably takes place chiefly through the nervous system, as in rabies. This view is supported by Brunner, Bruschettini and others. Beck has described a peculiar degeneration in the motor cells of the cord, which is very pronounced on the periphery of the cell, being confined almost exclusively to the cell body, usually leaving the nucleus intact; this change is also very pronounced at the point of origin of the axis-cylinder, and in the axis-cylinder itself, and it would seem to prove his theory that the toxine travels from the original site of injury, along the nerve fibre upon which it acts, producing tetanic contractions and spasms of the muscles and parts adjacent to the parts of infection; until finally reaching the cord the poison affects the motor cells and ganglia of the anterior horns, when the convulsive movements no longer remain localized but become general, involving almost every muscle in the body, especially the diaphragmatic muscles.

Park says there is, in addition, undoubtedly a diffusion of the poison by means of the blood and lymph, which statements have been shown to be true, by the injection of these toxic-laden fluids into the lower animals, thereby producing tetanic convulsions.

Kitasato also found serious exudates of the pleura and pericardial cavities, as well as the blood of tetanic animals would, when transferred to other animals, cause tetanic convulsions. Tetanus toxine has also been demonstrated in urine, and we may safely say (in a well-developed case of tetanus) that every fluid in the body is surcharged with toxines of the dreaded disease, and the cerebro-spinal fluid, according to Stintzing, contains a much more active and concentrated toxine than does the other fluids of the body. Now, this fluid, being in intimate contact, as it is, with the cord, and (as all post-mortem findings show), the primary lesion in tetanus is in the motor-ganglia of the anterior horn; then the rational treatment of tetanus resolves itself finally into:

1. The withdrawal of the highly laden toxic cerebro-spinal fluid from direct contact with the cells and cords by means of lumbar puncture.

2. The replacement of this fluid by either the use of antitetanic serum or normal salt solution (preference being given to the former) in order to equalize brain and cord circulation, and to prevent the occurrence of such disagreeable symptoms as severe headache, dizziness, pain along the spinal column, etc.; often observe when too much fluid is either injected or removed from the spinal canal. With this rather brief resume, I will report the five following cases, three of which came directly under my treatment while house-surgeon at Harlem Hospital, the remaining two having been seen by me in consultation:

CASE 1.—P. M. B. C., male, age twelve. Admitted to Harlem Hospital June 2, 1902, with the following history: Six days ago received pistol-shot wound in palm of left hand. First symptoms appeared five days after injury, when patient had violent attack of dyspnea, followed by slight tremor of left hand and rigidity of muscles of neck and thorax; symptoms rapidly grew worse, convulsions more frequent, constantly increasing in severity. Contraction of muscles of abdomen and locking of jaws were the last symptoms to appear. T. P. R. on admission 98-104-22. Temperature rapidly rose in forty-eight hours to 106 degrees. Pulse 112, respiration 38, and the patient died in the most violent tetanic spasms, particularly of the muscles of abdomen and chest.

*Treatment:* Wound was thoroughly cleansed and swabbed out repeatedly with pure carbolic acid. Wet dressing applied. Bromides and chloral frequently administered. Forced nutrition and stimulation. Hot packs and prolonged hot baths. In fact, he received the useless empiric and symptomatic therapy followed in such cases. I report this case in contrast to cases two, three, four and five of my series, and the five other cases reported by various writers in the medical journals since the above outlined treatment was instituted July 12, 1902.

CASE 2.—M. T., male, age twelve. Admitted to Harlem Hospital July 12, 1902, with a history of blank-cartridge wound on left hand, received July 4th. Wound was treated at dispensary.

Six days after injury, child complained of stiffness of jaws, pain in the back of the neck and chest, slight convulsions.

Admitted to hospital the next day with following symptoms: Jaws firmly set, risus sardonicus present, muscles of abdomen in state of constant tonic contractions, also marked tonic contraction or the orbicularis palpebrarum—the sphincter muscle of the eyelid; there was marked contraction of the fingers of the affected hand, which was bird-claw in character. T. P. R. on admission 101-116-24. Temperature on third day reached 104 degrees, becoming normal on the tenth day.

*Treatment:* Under careful asepsis, lumbar puncture was made between the third and fourth lumbar vertebra, twenty minims of cerebrospinal fluid was withdrawn and eight c. c. of antitetanic serum injected into the spinal sub-arachnoidean space. The injection was done very slowly, requiring about ten minutes to empty the syringe. There was absolutely no ill effects attending this treatment, and, on the contrary, the patient's muscular spasms relaxed, and in the course of thirty minutes he fell into a gentle sleep, which lasted a few hours, when spasms began again more violent than before, especially severe in the muscles of the abdomen and back, the former being so tense as to assume the boat-shaped abdomen peculiar to cerebrospinal menigitis. This condition would last from about eight to ten minutes and then relax. Another puncture was then made, M. twelve of spinal fluid withdrawn, and eleven c. c. of antitanic serum injected, which was followed by rest, remission of contractions, and decidedly less pain, as in the first puncture.

From this time on the patient received two or three lumbar punctures daily, between the third and fourth lumbar vertebra for eight successive days, and there was withdrawn a total of 161 drops of spinal fluid and ninety-two c. c. of tetanus antitoxine injected. On July 26th patient sat up in bed for two hours, was out of bed July 28th, on July 29th could open mouth as normal, complete relaxation of all muscles. Discharged cured July 30, 1902.

CASE 3.—W. B., male, ten years old. Admitted to Harlem Hospital September 10, 1902. Present history: Seven days ago, while attempting to vault a fence, fell and cut left wrist on broken bottle, was treated at St. Luke's Dispensary, where several sutures were taken in the wound and a wet dressing applied. Five days after injury had severe abdominal pain, applied for admission to an up-town hospital and was refused. On the night of the sixth day after injury, the patient was seen by me at the request of his attending physician, at his home on One Hundred and Forty-first street, and the diagnosis of tetanus made. The parents refused to let the child go to the hospital that night, so I decided to do a lumbar puncture and withdrew 125 drops of spinal fluid and injected twelve c. c. of antitoxine. The incubation period of this case was five days, the abdominal contractions having set in on the fifth day. The symptoms in this case, as in Case 2, were those of Rotter's subdivision B, a grave case. Without going into detail, the treatment of the case was as follows:

September 9th, 125 drops spinal fluid withdrawn, 12 c. c. antitoxine injected.

September 10th, 200 drops spinal fluid withdrawn, 9 c. c. antitoxine injected.

September 11th, 250 drops spinal fluid withdrawn, 14 c. c. antitoxine injected.

September 12th, 300 drops spinal fluid withdrawn, 15 c. c. antitoxine injected.

September 13th, 300 drops spinal fluid withdrawn, 12 c. c. antitoxine injected.

September 15th, 280 drops spinal fluid withdrawn, 12 c. c. antitoxine injected.

Making a total of 1,455 drops of spinal fluid withdrawn and 74 c. c. antitoxine injected. The reaction after each treatment in this case was decidedly more positive than in Case 2, followed by an almost immediate cessation of convulsions, pain and muscular rigidity. Patient sat up in bed September 22d and was discharged from the hospital cured September 26th. Prognosis was in this case, if possible, more grave than in Case 2, yet recovery was most satisfactory, under injections of only seventy-four c. c. of antitoxine, eighteen c. c. less than in Case 2; but I withdrew 1,294 drops more of the highly toxic spinal fluid than in Case 2. In short, I believe (and the two succeeding cases will show) that the gratifying results obtained from this treatment are due to the withdrawal in as large amounts as possible of the septic spinal fluid and not to any specific action of the antitoxine.

CASE 4.—Henry F., negro, age forty, railroad section hand. While lifting steel rail fell on leg, badly lacerating calf; was treated with turpentine and bandaged up with cotton waste used by the road; when seen July 12, 1903, five days after the injury and six hours after the onset of the disease, patient's jaws were firmly set, there was marked rigidity of the muscles of the neck and thorax with occasional convulsions, which steadily increased both as regards frequency and severity, resulting finally in complete opisthotonos. Physical examination, negative excepting wound on leg, which was suppurating slightly, otherwise wound seemed in fairly good condition considering treatment. T. P. R., 101-130-40. Diagnosis was immediately made of tetanus, and according to Rotter's subdivision of tetanus, this case was a mild one in subdivision A, or a very severe one in subdivision B, at best a very severe case. Immediate treatment being imperative, and having no antitetanic serum on hand a normal salt solution was prepared, skin and parts in lumbar region was made aseptic; a needle was then introduced under careful aseptic precautions between the third and fourth lumbar vertebra, and one ounce of spinal fluid withdrawn, the latter being replaced by the same amount of normal salt solution. This treatment was repeated daily for seven days, and at one time I was able to withdraw two ounces of spinal fluid, which was the greatest amount withdrawn at any one time. After first week lumbar puncture was done every second and finally every third day, patient gradually improving under each treatment until the seventeenth day, when patient was discharged cured. No bacteriological examination was made in this case, as I was not prepared to take a specimen, but I am positive from the clinical symptoms the diagnosis was correct, and the case was one of genuine tetanus, and is a splendid demonstration of the necessity of removing the spinal fluid in such cases immediately and in as large amounts as ossible.

CASE 5.—L. M., age six years, white, female. Present history, August 12, 1903, child stuck rusty nail in left foot, passing completety through foot just about one inch above the junction of the second and third phalanges. Wound was cleansed and tied up with a rag soaked in turpentine, as wounds are generally treated in the country. Eight days after injury the child was seen by me in consultation with Dr. Walker, and the diagnosis of tetanus made. Having no antitoxine on hand, I again resorted to lumbar puncture, withdrawing half an ounce of spinal fluid (that being all I could possibly withdraw) and replaced same with six fluid drachms of normal salt solution. This treatment was continued each day for six successive days, then every second and finally every third day, when treatment was wholly discontinued, and on the twelfth day after lumbar puncture was begun, patient sat up in bed; muscles were still rigid but all spasms and pain had disappeared. On the fifteenth day the patient was allowed to sit up in a chair; on September 8th discharged patient cured.

In the course of the observation and treatment of these cases I came to the following conclusions:

1. The indications in the treatment of tetanus are very strong and positive for urgent and forced nutrition and stimulation, on account of the marked exhaustion and most rapid wasting which directly or indirectly seems to aid in producing a fatal termination.

2. The shock and collapse often seen in administering medullary narcotics is not due to the removal of the spinal fluid and the injection of another fluid, but is due to the physiological action of the cocaine so injected.

3. That tetanus is not the hopeless condition that it has been pictured in the past, for the bacillus producing the disease remains localized at the original point of injury and generates a toxine, which toxine is taken up by the blood-vessels, the lymph-vessels, and finally is carried along the nearest nerve branches, according to Stinzing, in the interstices of the perineurium and endoneurium to the spinal cord, and in this way all the fluids of the body are charged with the poison; but, on account of the intimate contact of the spinal fluid with the cord, and on account of its concentrated condition, drawing as it does from both the toxine gathered by the nervous system and the lymph-vessels, the sooner this fluid is withdrawn from the spinal canal, thereby preventing further action on the motor-cells, and we might say checking all symptoms of the disease, the better the patient's chance of recovery. This fluid has to be withdrawn as the symptoms demand, once, twice or thrice daily if indicated, and must also be replaced by some sterile solution, and as the true value of the antitetanic serum has never been determined I favor its use if it can be had, giving the patient the benefit of the doubt; but, in case it can not be secured, then use either the normal salt solution or a two per cent. boracic acid solution. This, supplemented by the forced feeding and proper regard for the original wound, gives promise to reduce to nil the now terrible death-rate of ninety-five per cent. in this disease.

Since Cases 1, 2 and 3 of my series were reported by Dr. W. H. Luckett, attending surgeon to Harlem Hospital, in the *New York Medical News* of April 18, 1903, the following cases have been reported treated by the spinal route: Dr. John B. Murphy, of Chicago, reports a case in the *Journal of American Medical Association*, treated July 13, 1904, by aspiration of the cerebrospinal fluid and injection of morphineucain and salt solution, with complete recovery. In speaking of this case he says: "I do not know whether the benefit was attributable to the extraction of the cerebrospinal fluid or to the injection of the solution, or both. The fact that the fluid withdrawn on the first, second and third days contained pus and after that none, would lead one to believe that possibly the diminution of pressure in the cerebrospinal cavity aided the fluid in overcoming the infection; just as in epidemic cerebrospinal meningitis repeated spinal punctures relieves the pressure and gives the greatest percentage of recovery." He further states "that the aspiration and injections might be repeated with greater frequency (in his case it was performed daily) there seeming to be no ill effects from the withdrawal of the fluids or injections of the solution."

Dr. Enrique Yaniz, physician to the Municipal Hospital, Santa Ysabea, of Cardenas Island, of Cuba, reports four cases of severe tetanus, three of which were treated by the intrarachidean method, all of which recovered; the fourth was treated by the subcutaneous injection of antitetanic serum and died.

Dr. A. Garwood, New Braunfels, Texas, reports a case in the

*Texas Medical News*, October, 1903, treated by the intraspinal injection of antitetanic serum with complete recovery, making in all nine cases treated by lumbar puncture and the withdrawal of the septic spinal fluid, with one hundred per cent. of recoveries, while the two cases of this series treated by the subcutaneous injection of antitanic serum both died, verifying a late editorial in the *Journal of American Medical Association*, which states that up to the present time ninety-five per cent. of the cases of tetanus treated by the subcutaneous injection of antitetanic serum have resulted fatally.

